# Comparison of postoperative clinical outcome in medial‐pivotal and gradually reducing radius design cruciate‐retaining total knee arthroplasty—A multicenter analysis of propensity‐matched cohorts

**DOI:** 10.1002/jeo2.12002

**Published:** 2024-01-19

**Authors:** Tsuneari Takahashi, Kazuhisa Hatayama, Masahiro Nishino, Hironari Hai, Yuichiro Yamada, Kosuke Suzuki, Katsushi Takeshita

**Affiliations:** ^1^ Department of Orthopedic Surgery Ishibashi General Hospital Shimotsuke Japan; ^2^ Department of Orthopedic Surgery Japan Community Health Care Organization Gunma Central Hospital Maebashi Japan; ^3^ Department of Orthopedic Surgery Hokusuikai Memorial Hospital Mito Japan; ^4^ Department of Orthopedic Surgery Toyokawa City Hospital Toyokawa Japan; ^5^ Department of Orthopedic Surgery Nagoya Kyoritsu Hospital Nagoya Japan; ^6^ Department of Orthopedic Surgery Seirei Hamamatsu Hospital Hamamatsu Japan; ^7^ Department of Orthopedic Surgery, School of Medicine Jichi Medical University Shimotsuke Japan

**Keywords:** cruciate retaining, knee osteoarthritis, medial pivot, total knee arthroplasty

## Abstract

**Purpose:**

To clarify differences in surgery duration, postoperative knee range of motion (ROM), anterior and posterior (AP) laxity, and Forgotten Joint Score (FJS) in patients undergoing medial‐pivot (MP) and GRADIUS cruciate‐retaining (CR) total knee arthroplasty (TKA) surgeries.

**Methods:**

We examined patients who underwent either MP or CR TKA at six different Japanese centres. Patients were propensity score matched for age, sex, and preoperative hip‐knee angle (HKA). We compared the groups' average surgery duration, postoperative knee ROM, AP laxity, and FJS 1 year after surgery.

**Results:**

There were 86 study patients: 43 MP and 43 CR TKA matched for age, sex, and preoperative HKA. The MP group enjoyed a significantly shorter surgery duration (89.1 ± 10.9 mins vs. 95.7 ± 12.0 mins, *p* = 0.0091) and significantly better postoperative knee flexion than the CR group (123.7 ± 9.1° vs. 115.3 ± 12.4°, *p* < 0.001). The MP had significantly smaller postoperative AP laxity with 30° of knee flexion than the CR group (3.4 ± 1.3 vs. 5.6 ± 2.2 mm, *p* < 0.001). Conversely, postoperative AP laxity with 90° of knee flexion was significantly *larger* for the MP group (3.6 ± 1.3 vs. 2.7 ± 1.9 mm, *p* = 0.0098). There were no between‐group differences in postoperative FJS.

**Conclusions:**

The MP group showed better postoperative knee flexion, midrange AP knee stability, and shorter surgery duration.

**Level of Evidence:**

Level III, retrospective comparative study.

**Abbreviations**
ADL: activities of daily living
AP: anterior and posterior
BMI: body mass index
CR: cruciate‐retaining
FJS: forgotten joint score
HKA: hip‐knee angle
MP: medial‐pivot
MS: medially stabilized
PCL: posterior cruciate ligament
PS: posterior‐stabilized
ROM: range‐of‐motion
TKA: total knee arthroplasty

## INTRODUCTION

Total knee arthroplasty (TKA) is an established procedure for alleviating pain and improving activities of daily living in patients with end‐stage knee osteoarthritis (KOA) [1]. Unfortunately, 20% of patients express dissatisfaction after TKA [2, 3]; mid‐flexion instability is considered a likely reason for dissatisfaction [4, 5]. Decreasing dissatisfaction after TKA is important as links exist between knee flexion and quality‐of‐life after TKA in Asian patients [6].

The medially stabilized (MS) prosthesis mimics the knee's natural motion by employing a ‘ball‐and‐socket’ construct that selectively constrains the medial femoral‐tibial articulation [7, 8]. Consequently, the lateral compartment's articulation is less congruent with the medial compartment during flexion, allowing the femur to roll back here, rather than within the medial compartment [9]. Despite its morphological characteristics, previous systematic reviews, and meta‐analyses compared MS designs to conventional bearing and were unable to reach a clear conclusion on the clinical performance of the MS knee replacement construct [10, 11].

TKA procedures that use a gradually reducing radius (GRADIUS) design were introduced to gradually reduce the femoral radius and facilitate a smooth transition from stability through a full range of motion (ROM) [12]. To date, no studies have examined differences in postoperative ROM between patients who undergo MP or GRADIUS cruciate‐retaining (CR) TKA surgeries. We sought to clarify whether postoperative ROM differed depending on the prosthesis shape in matched groups. With postoperative flexion ROM as the primary endpoint, we hypothesized that postoperative knee flexion in the MP group would be significantly better than in the CR group because of the sacrifice of the posterior cruciate ligament (PCL).

## METHODS

We examined Japanese patients with KOA and varus knee deformities who complained of continuous pain and function loss despite conservative treatment. All patients either underwent MP (EVOLUTION, MicroPort Orthopedics, Inc) or CR TKA (ATTUNE, DepuySynthes) surgeries between August 2019 and December 2021. We ultimately identified 240 consecutive patients who underwent TKA at six different centres in five Japanese prefectures. Surgeries were performed according to the manufacturer's official instructions. Experienced knee surgeons determined the need for surgery based on clinical (e.g., a loss of ROM) and radiological findings (e.g., Kellgren and Lawrence classification grade 3 or 4) [13]. Prostheses were selected at each surgeon's discretion. We excluded patients with a previous TKA, knee osteotomy, or anterior (ACL) or PCL reconstruction due to the confounding effects of prior surgery on postoperative ROM. We recorded each patient's age, sex, body mass index (BMI), hip‐knee angle (HKA; varus indicates plus), pre and postoperative ROM for extension and flexion using a double‐armed goniometer, postoperative Forgotten Joint Score (FJS; worst, 0; best, 100) [14], and surgery duration.

### Surgical procedure

All TKAs were performed using a cemented, fixed‐bearing prosthesis. The ACL was dissected while the PCL was completely resected in the MP group and retained in the CR group. No patients underwent patella resurfacing. We performed a mechanically aligned TKA to achieve a neutral coronal mechanical limb alignment by cutting the femoral and tibial bones such that rectangular flexion and extension gaps were perpendicular to the mechanical axes. The distal femoral cut was made using an intramedullary alignment system. The posterior femoral cuts were made using a guide to establish the bony resection line parallel to the surgical epicondylar line and perpendicular to Whiteside's line [15]. The proximal tibial cut was made using an extramedullary alignment system. The definitive components were introduced with cement fixation.

### Clinical evaluations 1 year after surgery

**Figure 1:**
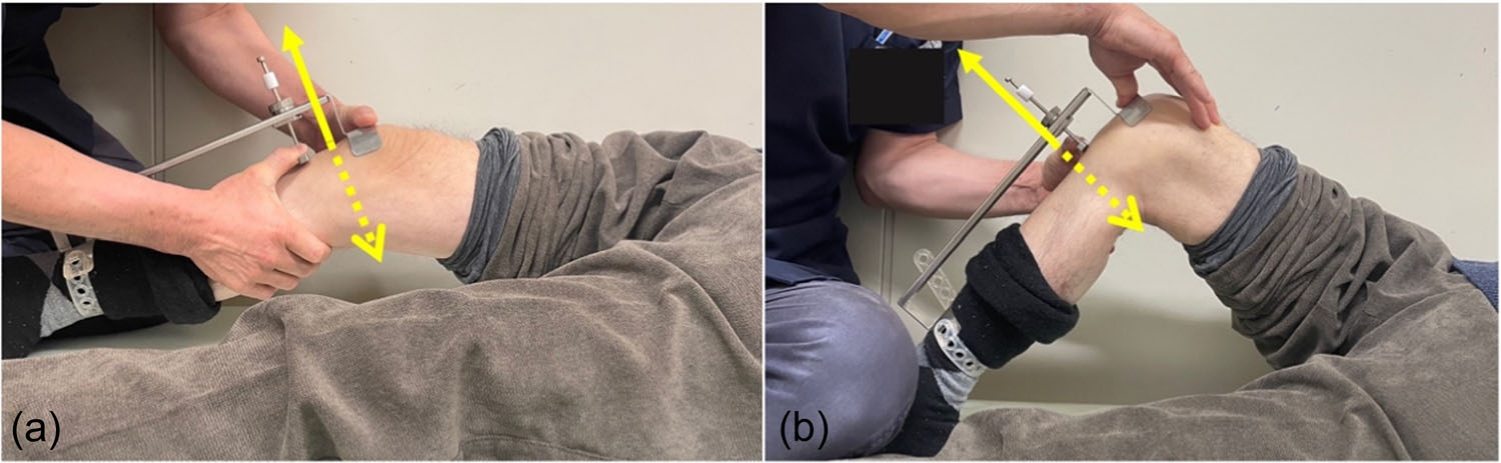
Postoperative knee AP laxity measurements made by using a Rolimeter at 30° (a) and 90° of knee flexion (b). The yellow arrow indicates anterior direction and the yellow dotted arrow indicates posterior direction of a manual max‐force to the tibia relative to the femur.

We measured postoperative extension and flexion ROM and FJS. Knee anterior and posterior (AP) laxity was measured with a Rolimeter (Aircast). Of note, the Rolimeter is cheap, easy to use, portable, and does not require radiological imaging [16]. The resultant measures were reliable and repeatable for the same and different observers [17] and between‐group comparisons were carried out. Each patient underwent the knee AP laxity measurement 1 year after surgery using Rolimeter during a routine follow‐up visit. The same knee surgeons in each centre performed the AP drawer test. Patients were subjected to the AP drawer test with 30° and 90° knee flexion three times. The AP laxity was calculated as the average of the three measurements (Figure 1).

### Statistical analysis

The groups were propensity score matched to minimize bias. Specifically, we employed single nearest‐neighbour matching (also known as one‐to‐one matching). Here, every MP case was matched with a CR case that most closely mirrored the observed patient's characteristics [18]. To control selection bias and ensure that covariates were balanced between the two groups, we matched cases to controls based on confounding variables such as age, sex, and preoperative HKA using propensity score calculated by logistic regression. With postoperative flexion ROM as the primary endpoint, the minimum sample size for an *α* error of 0.05, *β* error of 0.20, and Cohen's effect size of 0.8 was 52 patients (using G Power 3.1, Franz Paul) [19]. Based on the sample size with propensity matching, we assigned 43 patients to each group. The post hoc power analysis was sufficient with at least 95.6% power. Data are presented as means and standard deviations. All statistical analyses were performed using EZR software [20]. The sample size was calculated in advance based on the results of an unpaired t‐test for the primary outcome. The significance level was set at *p* < 0.05.

## RESULTS

We enroled 86 patients in this study: their demographics are shown in Table 1. Forty‐three patients in the MP group and CR group were matched for age (73.5 ± 5.2 years old in the MP group and 73.3 ± 5.2 years old in CR group), sex (female ratio; 83.7% in both MP group and CR group), and preoperative HKA (12.0 ± 4.9° varus in MP group and 12.1 ± 4.9° varus in CR group). There were no significant between‐group differences for BMI (27.1 ± 3.9 kg/m^2^ in the MP group and 25.5 ± 3.7 kg/m^2^ in the CR group), preoperative knee flexion (121.0 ± 13.7° in MP group vs. 119.2 ± 15.4° in CR group) and extension (−7.5 ± 6.2° in MP group vs. −8.7 ± 6.5° in CR group).

**Table 1:** Group‐wise comparison of patients' pre‐, peri‐, and postoperative characteristics.

Variables	MP group (n = 43)	CR group (n = 43)	*p* Value^a^
	Mean (SD)	Mean (SD)	
Patients' preoperative characteristics			
Age (years)	73.5 (5.2)	73.3 (5.2)	>0.05
Sex (female)^b^	83.7 (%)	83.7 (%)	>0.05
Preoperative HKA (°)	12.0 (4.9)	12.1 (4.9)	>0.05
BMI (kg/m^2^)	27.1 (3.9)	25.5 (3.7)	>0.05
Preoperative ROM for flexion (°)	121.0 (13.7)	119.2 (15.4)	>0.05
Preoperative ROM for extension (°)	−7.5 (6.2)	−8.7 (6.5)	>0.05
Surgical time (mins)	89.1 (10.9)	95.7 (12.0)	0.0091
Postoperative ROM for flexion (°)^c^	123.7 (9.1)	115.3 (12.4)	<0.001
Postoperative anterior‐posterior laxity^c^			
30° of knee flexion (mm)	3.4 (1.3)	5.6 (2.2)	<0.001
90° of knee flexion (mm)	3.6 (1.3)	2.7 (1.9)	0.0098
Postoperative FJS^c^	72.2 (25.4)	76.2 (17.4)	>0.05

Abbreviations: BMI, body mass index; FJS, Forgotten Joint Score; HKA, hip knee angle, ROM, range of motion; SD, standard deviation.

^a^ *χ* ^2^ tests for sex; Student's *t* test for other numerical variables.

^b^ Presented as the number and percentage of patients.

^c^ Assessments 1 year after surgery.

**Figure 2:**
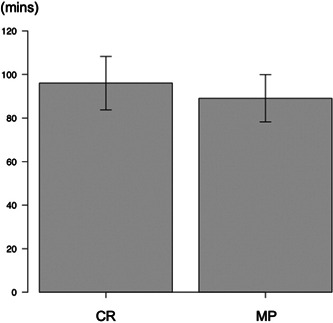
Comparison of surgery duration between the groups. CR, cruciate‐retaining; MP, medial‐pivot.

**Figure 3:**
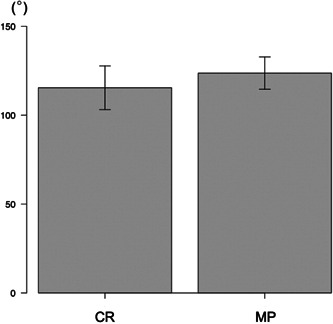
Comparison of postoperative knee flexion between the groups. CR, cruciate‐retaining; MP, medial‐pivot.

The MP group had a significantly shorter surgery duration than the CR group (89.1 ± 10.9 mins vs. 95.7 ± 12.0 mins, *p* = 0.0091) (Figure 2). Postoperative knee flexion in the MP group was significantly better than in the CR group (123.7 ± 9.1° vs. 115.3 ± 12.4°, *p* < 0.001) (Figure 3). In addition, the MP group had significantly smaller postoperative AP laxity with 30° of knee flexion than the CR group (3.4 ± 1.3 vs. 5.6 ± 2.2 mm, *p* < 0.001). On the other hand, postoperative AP laxity with 90° of knee flexion was significantly larger in the MP than CR group (3.6 ± 1.3 vs. 2.7 ± 1.9 mm, *p* = 0.0098). There were no between‐group differences in postoperative FJS (72.2 ± 25.4 in the MP group vs. 76.2 ± 17.4 in the CR group) (Table 1).

## DISCUSSION

In the MP group, surgery duration was shortened by an average of 6.6 min over the CR group. Postoperative knee flexion in the MP group was significantly better (8.4°) than in the CR group. In addition, postoperative AP laxity with the mid‐flexion angle in the MP group was significantly smaller—averaging 2.2 mm—than in the CR group.

Recently Kato et al. described the clinical superiority of MS designs to conventional posterior‐stabilized (PS) bearing [21]. They reported the MP group exhibited a significantly better FJS than the PS group. In addition, postoperative knee flexion and extension 1 year later were similar in both groups. These differ from our results. The control group's choice of prosthetic might explain observed differences in postoperative FJS and knee flexion. They used the Attune PS rotating platform as the control group, whereas we used the Attune CR fixed bearing as a control group. No studies have directly compared the postoperative ROM between Attune PS rotating platform and Attune CR fixed bearing. Past studies reported postoperative ROM for flexion after TKA using the Attune CR fixed bearing [22, 23] and their results were similar to ours. Arbuthnot et al. compared 168 patients (232 knees) who had undergone TKA with either a PS or CR prosthesis and concluded that ROM was superior in the PS group and that the PS prosthesis for TKA provided more reliable outcomes with a better maximum flexion than a CR prosthesis [24]. Ritter et al. described that PCL excision in CR TKA resulted in better knee flexion [25]. The potential effects of PCL retention on knee joint proprioception remain controversial.

Warren et al. found that the PCL‐retaining prosthesis was associated with greater improvements in postoperative proprioception than the PCL‐sacrificing design [26]. On the other hand, Swanik et al. described that PCL retention did not significantly improve proprioception [27]. Therefore, future studies should use dynamic sensory evaluation methods to determine potential knee joint differences between the MP and CR groups.

The CR surgery duration was likely prolonged by the need to create an adequate flexion gap while retaining the PCL. Importantly, the PCL must be maintained under appropriate tension to realize the kinetic benefits of its retention [28]. PCL release [29], resection of additional tibial bone to increase the posterior tibial slope [30], and sizing down the femoral component can help achieve an adequate flexion gap. In CR TKA, these additional procedures may have prolonged the surgery duration.

Finally, postoperative AP laxity with 30° knee flexion was significantly smaller in the MP group than in the CR group (though larger with 90° knee flexion). These differences were believed to be due to differences in insert design and PCL sacrifice/retention. Ishii et al. described that postoperative AP laxity was significantly higher for the CR (compared to PS) TKA at 30° [31]. In addition, an MS prosthesis had better sagittal stability [32]. Thus, implant design and conformity appear to affect mid‐flexion sagittal stability after TKA more than the presence or absence of the PCL. MP TKA provided good mid‐flexion sagittal stability and a better flexion angle than CR TKA. On the other hand, 1 year after surgery, the AP laxity at 90° knee flexion was smaller in the CR group. The PCL in the CR group might have been tighter than in the MP group, resulting in a smaller flexion ROM after surgery.

### Limitations

Our study's results should be considered within the context of specific limitations. However, the groups were propensity‐score matched to reduce bias. Patients underwent their respective surgeries at six different centres in Japan; this likely introduced bias as different surgeons performed the various surgeries. However, multicenter comparisons accurately reflect general practice patterns and potentially reduce bias introduced by single‐centre designs. In addition, surgeons from all six centres held consensus meetings and adopted a uniform technique. In addition, we excluded treatment failures from our outcome analysis. This likely introduced selection bias; however, by eliminating treatment failures were avoided their confounding effects on TKA outcomes. Although this study reported short‐ to medium‐term outcomes, additional, long‐term follow‐up studies are needed to draw meaningful comparisons among the various TKA treatment models.

## AUTHOR CONTRIBUTIONS

The conception and design of the study were done by Tsuneari Takahashi and Kazuhisa Hatayama. The acquisition of data was taken care of by Tsuneari Takahashi and Kazuhisa Hatayama, Masahiro Nishino, Hironari Hai, Yuichiro Yamada, and Kosuke Suzuki. Analysis and/or interpretation of data was carried out by Tsuneari Takahashi and Kazuhisa Hatayama. The drafting of the article was done by Tsuneari Takahashi, Kazuhisa Hatayama, Yuichiro Yamada, and Katsushi Takeshita. Revising the article critically for important intellectual content was taken care of by Katsushi Takeshita. All authors have contributed significantly to the study, approved the article, and agreed with the submission.

## CONFLICT OF INTEREST STATEMENT

This study was funded by a research grant from MicroPort Orthopedics.

## ETHICS STATEMENT

This study was conducted following the principles of the Declaration of Helsinki. Our Institutional Ethics Committee approved the study (Date: September 24, 2020, Number: 20‐037).

## Data Availability

Data will be made available on request.
